# Effects of the Informed Health Choices podcast on the ability of parents of primary school children in Uganda to assess the trustworthiness of claims about treatment effects: one-year follow up of a randomised trial

**DOI:** 10.1186/s13063-020-4093-x

**Published:** 2020-02-14

**Authors:** Daniel Semakula, Allen Nsangi, Andrew D. Oxman, Matt Oxman, Astrid Austvoll-Dahlgren, Sarah Rosenbaum, Angela Morelli, Claire Glenton, Simon Lewin, Laetitia Nyirazinyoye, Margaret Kaseje, Iain Chalmers, Atle Fretheim, Christopher J. Rose, Nelson K. Sewankambo

**Affiliations:** 1 College of Health Sciences, Makerere University, Kampala, Uganda; 2 University of Oslo, Oslo, Norway; 3 Centre for Informed Health Choices, Norwegian Institute of Public Health, Postboks 222, Skøyen, 0213 Oslo, Norway; 4 Infodesignlab, Oslo, Norway; 5 Health Systems Research Unit, South African Medical Research Council, Cape Town, South Africa; 6 School of Public Health, College of Medicine and Health Sciences, University of Rwanda, Kigali, Rwanda; 7 Tropical Institute of Community Health, Kisumu, Kenya; 8 James Lind Initiative, Oxford, UK

**Keywords:** Podcast, Health choices, Treatment effects, Evidence-informed decision-making, Critical thinking, Health literacy, Evidence-based health care, Informed health choices, Claims about treatment effects

## Abstract

**Introduction:**

Earlier, we designed and evaluated an educational mass media intervention for improving people’s ability to think more critically and to assess the trustworthiness of claims (assertions) about the benefits and harms (effects) of treatments. The overall aims of this follow-up study were to evaluate the impact of our intervention 1 year after it was administered, and to assess retention of learning and behaviour regarding claims about treatments.

**Methods:**

We randomly allocated consenting parents to listen to either the Informed Health Choices podcast (intervention) or typical public service announcements about health issues (control) over 7–10 weeks. Each intervention episode explained how the trustworthiness of treatment claims can be assessed by using relevant key concepts of evidence-informed decision-making. Participants listened to two episodes per week, delivered by research assistants. We evaluated outcomes immediately, and a year after the intervention. Primary outcomes were mean score and the proportion with a score indicating a basic ability to apply the key concepts (> 11 out of 18 correct answers) on a tool measuring people’s ability to critically appraise the trustworthiness of treatment claims. Skills decay/retention was estimated by calculating the relative difference between the follow-up and initial results in the intervention group, adjusting for chance. Statistical analyses were performed using R (R Core Team, Vienna, Austria; version 3.4.3).

**Results:**

After 1 year, the mean score for parents in the intervention group was 58.9% correct answers, compared to 52.6% in the control (adjusted mean difference of 6.7% (95% CI 3.3% to 10.1%)). In the intervention group, 47.2% of 267 parents had a score indicating a basic ability to assess treatment claims compared to 39.5% of 256 parents in the control (adjusted difference of 9.8% more parents (95% CI 0.9% to 18.9%). These represent relative reductions of 29% in the mean scores and 33% in the proportion of parents with a score indicating a basic ability to assess the trustworthiness of claims about treatment effects.

**Conclusions:**

Although listening to the Informed Health Choices podcast initially led to a large improvement in the ability of parents to assess claims about the effects of treatments, our findings show that these skills decreased substantially over 1 year. More active practice could address the substantial skills decay observed over 1 year.

**Trial registration:**

Pan African Clinical Trial Registry (www.pactr.org), PACTR201606001676150. Registered on 12 June 2016.

## What is already known?

In a trial conducted in 2016, the Informed Health Choices podcast was effective in improving people’s ability to critically assess the trustworthiness of claims about treatment effects immediately after the intervention.

## What are the new findings?

The effect of the Informed Health Choices podcast on people’s ability to appraise the trustworthiness of claims about treatment effects reduced markedly in the year after implementation of the intervention, indicating a substantial skills decay.

## What do these findings imply?

The effect of the Informed Health Choices podcast on people’s ability to think critically about claims about the effects of treatments likely reduces markedly with time, in the absence of additional intervention or regular practice. In order for learning to be sustained, considerations should be made to reinforce the messages of the podcast.

## Background

Many countries and societies today are faced with an overabundance of claims (things people say) about the effects of treatments and advice about what we should do to improve or maintain our health [1–6]. Some of these are about the effects of medical or surgical interventions, preventative or palliative individual and public health interventions. These claims have increased in frequency, geographical reach and speed of spread as access to information, the Internet and social media use increases [7–10]. Many of these claims are not based on trustworthy evidence [11–14] and represent a portion of what some people term as “fake” health news, advice or stories. Many people do not have the required aptitude to critically appraise the trustworthiness of claims about the effects of treatments, and often act on them in making choices about treatments [15–24]. Poorly informed health choices can result in overuse of ineffective or harmful treatments (actions intended to maintain or improve the health of individuals or communities), underuse of effective treatments, waste and unnecessary suffering [25–29]. Making well-informed choices about treatments is especially important in low-income countries, which have few resources to waste and where the repercussions for making poor health choices are likely to be greater [30–34]. However, there are few resources to teach people without a health and/or research background to think more critically in evaluating claims about treatments and few studies have evaluated the effects of interventions to teach patients or the public to think critically about health choices [35, 36]. As part of the Informed Health Choices (IHC) project [37], we developed a mass media intervention (an edutainment podcast) to help fill this gap.

We began by identifying key concepts that people must understand and apply when assessing claims about treatments [38, 39]. We call these the informed health choices (IHC) key concepts. Together with journalists in Uganda, we assessed which key concepts are most important for the public to understand [40]. Our mass media intervention was developed to teach 9 of the now 49 IHC key concepts (Table 1) to parents of primary school children [41].

**Table 1:** Key concepts included in the IHC mass media (podcast) and primary school resources

Included in both the IHC mass media and primary school resources	Included in the IHC mass media resources (podcast) only	Included in the IHC school resources only
Treatments may be harmful. People often exaggerate the benefits of treatments and ignore or downplay potential harms. However, few effective treatments are 100% safe (included in podcast episode 1)		
Personal experiences or anecdotes (stories about how a treatment helped or harmed someone) are an unreliable basis for predicting the effects of most treatments (included in podcast episode 3)		
	A treatment outcome may be associated with a treatment, but not caused by the treatment. The fact that a possible treatment outcome (i.e. a potential benefit or harm) is associated with a treatment does not mean that the treatment caused the outcome. The association or correlation could instead be due to chance or some other underlying factor. For example, people who seek and receive a treatment may be healthier and have better living conditions than those who do not seek and receive the treatment. Therefore, people receiving the treatment might appear to benefit from the treatment, but the difference in outcomes could be because they are healthier and have better living conditions, rather than because of the treatment (included in podcast episode 4)	
How widely or how long a treatment is used is not a reliable indicator of how beneficial or safe it is. Treatments that have not been properly evaluated but are widely used or have been used for a long time are often assumed to work. Sometimes, however, they may be unsafe or of doubtful benefit (included in podcast episode 5)		
		New, brand-named, technologically impressive, or more expensive treatments may not be better than available alternatives
Opinions of experts or authorities do not alone provide a reliable basis for deciding on the benefits and harms of treatments. Doctors, researchers, patient organisations and other authorities often disagree about the effects of treatments. This may be because their opinions are not always based on systematic reviews of fair comparisons of treatments (included in podcast episode 6)		
		Conflicting interests may result in misleading claims about the effects of treatments. People with an interest in promoting a treatment (in addition to wanting to help people) - for example, to make money - may promote treatments by exaggerating benefits, ignoring potential harmful effects, cherry picking which information is used, or making false claims. Conversely, people may be opposed to a treatment for a range of reasons, such as cultural practices
Comparisons		
Evaluating the effects of treatments depends on making appropriate comparisons. If a treatment is not compared to something else, it is not possible to know what would happen without the treatment, so it is difficult to attribute outcomes to the treatment (included in podcast episode 2)		
Comparisons of treatments must be fair. Apart from the treatments being compared, the comparison groups need to be similar at the beginning of a comparison (i.e. “like needs to be compared with like”) (included in podcast episode 7)		
		If possible, people should not know which of the treatments being compared they are receiving. People in a treatment group may behave differently or experience improvements or deterioration as a result of knowing the treatment to which they have been assigned. If this phenomenon is associated with an improvement in their symptoms it is known as a placebo effect; if it is associated with a harmful effect it is known as a nocebo effect. If individuals know that they are receiving a treatment that they believe is either better or worse than an alternative (that is, they are not “blinded”), some or all of the apparent effects of treatments may be due either to placebo or nocebo effects
		Small studies in which few outcome events occur are usually not informative and the results may be misleading. When there are few outcome events, differences in outcome frequencies between the treatment comparison groups may easily have occurred by chance and may mistakenly be attributed to differences in the effects of the treatments
The results of single comparisons of treatments (trials) can be misleading. A single comparison of treatments rarely provides conclusive evidence and results are often available from other comparisons of the same treatments. These other comparisons may have different results or may help to provide more reliable and precise estimates of the effects of treatments (included in podcast episode 8)		
Choices		
Because treatments can have harmful effects as well as beneficial effects, decisions should not be based on considering only their benefits. Rather, they should be informed by the balance between the benefits and harms of treatments. Costs also need to be considered (included in all episodes)		

*IHC* Informed Health Choices

The concepts are shown here as they are described in the key concepts list, [38, 39]

### Description of the intervention (the Informed Health Choices podcast)

We developed pre-recorded audio messages with teachings about critically appraising the trustworthiness of claims about the effects of treatments. The podcast had 13 episodes in both English and Luganda, a local language widely spoken in the study area: an introduction to the series; eight main episodes; three short recap episodes, each of which summarised two of the first six main episodes; and a conclusion. Each of the eight main episodes included a short story with an example of a treatment claim, an explanation of the IHC key concept applied to the claim, and another example within the same story illustrating the concept. The examples of claims were identified from scanning recent mass media reports and interviewing parents. We also gave the parents a checklist summarising the key messages in the podcast and a song (the IHC theme song) to reinforce the messages of the podcast [42]. The podcast is available online at https://www.youtube.com/watch?v=_QVdkJIdRA8&list=PLeMvL6ApG1N0ySWBxPNEDpD4tf1ZxrBfv.

As described in the paper describing the initial assessment results, the research assistants delivered the intervention to parents on multimedia players in the patients’ workplaces and/or homes over a period of 7–10 weeks. They listened to two new episodes each week and a recap of the previous episodes. Following this observed listening, they were given the content of the podcast on portable multimedia players to listen on their own before they completed the evaluation tool [43]. This information has been presented before [43]. Very much aware of self-plagiarism, we only present it here for purposes of making it easier for the reader, in case one may not be able to find it easily in previous publications.

In 2016, we conducted a randomised trial to evaluate the effects of the IHC podcast on the ability of parents in Uganda to apply key concepts of evidence-informed decision-making in appraising the trustworthiness of claims about the effects of treatments. The trial showed that parents who listened to the IHC podcast had a large improvement in their ability to assess treatment effects shortly after listening to all of the episodes [43]. We also developed learning resources to teach 12 of the key concepts (Table 1) to children in the fifth year of primary school in Uganda. A linked cluster-randomised trial showed that the IHC primary school intervention also had a large effect on the ability of the children to apply those IHC key concepts [44].

Follow up was for 7–10 weeks and after 1 year. In this report we present methods and results of a 1-year follow-up study of the effects of an educational podcast. The main aim of the follow-up study was to assess parents’ ability to assess the trustworthiness of claims about the effects of treatments a year after listening to the podcast. This would enable us to determine how much of the critical appraisal skills learned were retained overall, and for each IHC key concept. Many clinical trials have short follow-up periods and are implemented in highly controlled environments with highly selective outcomes prespecified by investigators. Although follow-up studies can be logistically challenging, they can provide valuable information about the longer-term effects (benefits and harms) and costs of health interventions that investigators were not able to obtain during the initial trial follow-up period. We also aimed to assess if and how parents were able to apply their newly learned key concepts in making decisions about treatments in the year following the intervention, and their intended behaviours going forward. The results of the sister studies - the 1-year follow-up study of the primary school resources and process evaluations for the podcast and the primary school resources - are reported in companion articles elsewhere [45–47].

## Methods

This was a follow-up assessment to a parallel-group randomised trial comparing the IHC podcast for teaching critical appraisal skills to a series of recordings designed to sound like typical public service announcements about health issues. Details on the study methods can also be found in the trial protocol [48] and the report of the initial results [43]. Some of the information in this section has been presented in some form in our earlier publications [43, 48]. We reuse it here only for purposes of providing clarity to the reader who may have difficulties accessing the information from our previous publications, well aware of the concept of self-plagiarism. We have done our best to acknowledge and reference appropriately.

### Eligibility

Parents in the 1-year follow-up study were those who participated in the randomised trial that evaluated the impact of the IHC podcast in 2016 [43]. To participate in that study, parents had to understand English or Luganda and provide written consent. Parents who were unable to hear or were not contactable by telephone, health researchers and participants in the development of the podcast were excluded. Parents of children who participated in the development of the primary school resources were also excluded.

### Participants

The study was conducted in central Uganda. As reported previously [43, 48], we recruited parents and guardians of children in the fifth year of primary school who were participating in the IHC primary school intervention trial [44]. Parents were recruited from both intervention and control schools. We recruited a convenience sample of participants at parent meetings held at 20 intervention schools and 15 control schools, between late August and early November 2016. Of the 675 parents who consented and were randomised, 561 (83%) completed the test used to measure their ability to assess claims about the effects of treatments shortly after listening to the podcast, in 2016. We attempted to follow up all 561 parents 1 year after they completed the test. We contacted those who were still reachable by phone and asked them to complete the test again.

### Randomisation and masking

We stratified the parents by highest level of formal education attained (primary school, secondary school or tertiary education) and the allocation of their children’s school in the trial of the primary school resources (intervention or control). We generated randomisation sequences with block sizes of four and six with equal allocation ratios within each block, using www.sealedenvelope.com. A statistician who was not a member of the research team generated the allocation sequence, and together with his team prepared six randomisation lists (one for each combination of the two stratification variables) with unique codes. They labelled opaque envelopes with the unique codes, inserted slips of paper with the study group allocated to each code and sealed them. We allocated groups of participants at the end of each day on which a meeting was held. Upon return to the trial management office, the research assistant responsible for allocation opened the next available envelope in the stratum corresponding to each parent’s education level and whether the child of that parent went to a school in the intervention or control arm of the primary school resources trial.

The research assistants who delivered the podcast, the principal investigators supervising them (DS and AN), the study participants and the statistician analysed the data all knew whether the participants received the IHC podcast or the public service announcements. To ensure uniform performance in delivery of the podcast and the public service announcements and in the assessment of outcomes, all study staff were trained before the start of the trial and received refresher training during the trial.

### Procedures

Participants could choose whether they wanted to listen to the podcast or the announcements in English or Luganda. Participants in the control group listened to typical public service announcements about the same conditions that were used in the IHC podcast. The podcast and the public service announcements were produced in collaboration with a Ugandan radio producer and actors. Research assistants helped with recruitment, delivery of the podcast, follow up, and administration of the test used as the outcome measure. They delivered episodes of the podcast or the public service announcements to the participants over a period of 7–10 weeks. To ensure that the participants listened to each episode (or announcement), the research assistants visited each participant once per week, delivering two episodes via a portable media player and speaker. In addition to listening to the episodes delivered by the research assistants, we provided participants with the complete podcast and the IHC theme song on MP3 players, so that they could replay them at their convenience.

The test included 18 multiple-choice questions from the Claim Evaluation Tools database [49–51] - two for each of the nine IHC key concepts (Additional file 1). Because many parents did not have English as their first language and many had poor reading skills, we developed a Luganda audio version of the test to be administered by an interviewer [52]. We were careful to ensure that the examples used in the questions were different from what was used in the podcast, and that participants would be able to understand the language that was used without having listened to the podcast. For the 1-year follow up, participants answered the same 18 questions that they answered initially. Research assistants visited the participants individually and administered the tests.

The questions had between two and four response options, with an overall probability of answering 37% of the questions correctly by chance alone. We used an absolute (criterion-referenced) standard to set a cutoff for a passing score (11 out of 18 questions (61%) answered correctly) and a mastery score (15 out of 18 questions (83%) answered correctly) [53].

There were 8 additional multiple-choice questions included, making 26 questions in total. These questions addressed four IHC key concepts not covered by the podcast (Table 1). They were included because the same test was used in the linked randomised trial evaluating the primary school resources, and those IHC key concepts were covered in the primary school resources [44]. Responses to these eight questions were not included in the primary analyses of the podcast trial. The test also included questions that assessed intended behaviours and self-efficacy.

We calculated retention of what was learned by parents in the podcast group to help interpret the results. Retention is reported as the test scores in the podcast group after 1 year relative to their test scores shortly after listening to the podcast. Retention for the mean score is adjusted for chance, by subtracting the probability of answering questions correctly by chance (37%) from the means. These analyses were not specified in the protocol, but we decided to conduct them to help interpret the results.

In the test taken after 1 year, we also collected data on self-reported behaviours. We made the comparisons shown in Tables 2, 3 and 4, with the hypotheses shown in Table 2. These also were not specified in the original protocol for the trial but were planned prior to collecting the 1-year follow-up data.

**Table 2:** Comparisons related to self-reported behaviours in the 1-year follow up

Question	Hypothesis and basis for the hypothesis
How often do you hear treatment claims?	Children in the intervention group will report hearing treatment claims more often because they are more aware of treatment claims and identifying them when they are made
[For the last treatment claim that you heard], did you consider whether to believe the basis of that treatment claim?	A larger proportion of children in the intervention group will answer yes because of being more aware that many claims do not have a reliable basis
How sure are you that the treatment claim you heard is true or can be trusted?	A smaller proportion of children in the intervention group will answer “very sure” or “I don’t know”, and a larger proportion of children in the intervention group will answer this question consistently with their answer to the preceding question about the basis of the claim (Table 3) because they are better able to assess the trustworthiness of claims and know that many claims do not have a reliable basis
How sure are you about the advantages and disadvantages of the [most recent] treatment you used?	A larger proportion of the children in the intervention group will answer “not very sure because I only know about the advantages”. A smaller proportion will answer “very sure”, because information about the disadvantages of treatments is often lacking. However, this difference, if there is one, will likely be small, because children in the intervention group are more likely to consider and seek information about the disadvantages of treatments
Who do you think should decide for you whether you should use a treatment or not use a treatment?	A larger proportion of the children in the intervention group will answer that they want to be included (A, C, D, F or G) because they have learned about how to make informed health choices; and that someone who knows a great deal about treatments should be included (E, F or G), because of being more aware of the importance of assessing the reliability of evidence of effects and the skills needed to do this. However, this difference, if there is one, will likely be small, because children in the intervention group are more likely to recognise that expert opinion alone is not a reliable basis for a claim about treatment effects
	A larger proportion of children in the intervention group will answer, “Not very sure because there was not a good reason behind the claims about the advantages of the treatment”, because they are more likely to identify a claim with an unreliable basis
Given your thoughts about the basis of the claim, what did you decide to do about the treatment?	A smaller proportion of children in the intervention group versus the control group would choose to use a treatment (in question 29.7), having recognised that the basis for the claim was untrustworthy (in question 29.6)

**Table 3:** Consistent (correct) answers regarding certainty about treatment claims^a^

If you heard about a treatment claim, what was its basis?	How sure are you that the treatment claim you heard is true or can be trusted?
Someone’s personal experience using the treatment	Not very sure because the reason behind the claim was not good
What an expert said about it	Not very sure because the reason behind the claim was not good
A research study that compared the treatment with another treatment or no treatment	Not very sure because the reason behind the claim was not good OR Very sure because the reason behind the claim was good
Something else	Not very sure because the reason behind the claim was not good
I could not tell what the treatment claim was based on	Not very sure because I don’t know the reason behind the claim

^a^Questions 28.5 and 28.6 in Additional file 1

**Table 4:** Exclusion criteria for self-reported behaviours

Response options for questions 28.2 and 29.3	Response to questions 28.3 and 29.4
28.2 What treatment claim did you last hear about?	28.3 Please write down the claim that you last heard
29.3 What was the treatment for which you or an adult made the decision?	What was the claim about the treatment for which you or an adult made the decision?
Using a medicine (e.g. taking a tablet or syrup)	Exclude, if the claim is not about a medicine
Getting an operation (e.g. removing a bad tooth)	Exclude, if the claim is not about an operation
Using something to feel better or to heal more quickly (e.g. using a bandage or glasses)	Exclude, if the claim is not about equipment
Something else (eating food or drinking something to feel better (e.g. herbs or fruit))	Exclude, if the claim is not about eating/drinking something e.g. herbs or fruit
Avoiding doing something to feel better (e.g. not drinking milk)	Exclude, if the claim is not about avoiding something
Something else	Exclude, if the claim is not about a treatment (“anything done to care for yourself, so you stay well or, if you are sick or injured, so you get better and not worse”)

The trial employed 29 research assistants, each of whom was allocated up to 25 participants to follow up and deliver the interventions to. They were allocated either control or intervention participants but not both. The research assistants kept logs, including reasons for dropping out, and they recorded any unexpected adverse events. We also collected in-depth qualitative data from interviews and focus group discussions on potential adverse effects in the process evaluation [46].

The investigators conducted the follow-up assessment, with the help of research assistants. Given the nature of the intervention it was not possible to blind the outcome assessors.

### Outcomes

The primary outcomes were:

1. Mean score (percent of correct answers) on the test taken a year after listening to all the podcast episodes or all the public service announcements
2. Proportion of participants with a score indicating a basic understanding and ability to apply the key concepts

Secondary outcomes were:

1. Retention of what was learned
2. Proportion of participants with a score indicating mastery of the concepts
3. for each IHC key concept, the proportion of participants answering both questions correctly
4. Intended behaviours and self-efficacy
5. Self-reported behaviours
6. Mean scores for the parents whose children were included in the intervention arm of the trial of the IHC primary school resources (to assess any effect of having a child in the intervention arm of a related trial teaching children the same concepts)

### Statistical analysis

We estimated that 397 participants were needed to detect an improvement of 10% in the podcast group based on a method described by Donner et al. [54], as described previously [43]. Allowing for a 20% loss to follow up, we estimated that we would need a sample size of 497 participants. Participants’ data were analysed per their allocation group (intention to treat).

For the primary and secondary outcomes, we modelled the two stratification variables (education level and child’s school allocation in the IHC primary school trial) as fixed effects, using logistic regression for dichotomous outcomes and linear regression for continuous outcomes. Missing values were counted as wrong answers. For intended behaviours and self-efficacy, we dichotomized each outcome by combining categories, for example (1) “very likely” with “likely” and (2) “very unlikely”, “unlikely” and “don’t know” with missing responses. We reported the proportion in each category and in the combined categories (“likely or very likely” in this example).

For comparisons of how frequently participants reported hearing treatment claims, we analysed the ordinal data using ordinal logistic regression. We also dichotomised the responses (one claim or more most days or most weeks versus most months, almost never, do not know or missing), which we analysed using logistic regression. We dichotomised the responses for the other comparisons (Table 2).

Because these questions were not previously validated, we used open-ended questions to validate the answers to the preceding question about the type of treatment and to validate that they understood what a treatment claim is. We coded answers to these questions as correct or incorrect and excluded all the participants who did not correctly identify the type of treatment (Table 4) or who did not report a treatment claim, from the comparisons in Table 2. We also excluded participants who responded: “I have never heard of any treatment claims.” For the comparisons about a claim about a treatment for which they made a decision, we excluded participants who responded: “I have never decided to use or not to use a treatment.” We assessed the consistency of answers by matching participants’ responses with the basis of the treatment claim as shown in Table 3. Additionally, we developed exclusion criteria for consistent responses across behaviour-related questions as outlined in Table 4 below.

To explore the risk of bias due to attrition, which was larger in the control group than in the podcast group, we conducted two sensitivity analyses. First, we calculated Lee’s treatment effect bounds [55] on the mean difference in test scores, which provides worst-case and best-case estimates of the difference in test scores under extreme assumptions about the effect of possible non-random attrition. To achieve this, we calculated upper and lower bounds for the mean difference in test scores. The bounds are constructed by trimming the group with less attrition at the upper and lower tails of the outcome (test score) distribution, respectively. In this analysis, the sample was trimmed in the podcast (intervention) group so that the proportion of parents included in the analysis was equal for both groups. We did not adjust for covariates in this analysis. Second, we reanalysed the results for the primary outcomes for the initial test, excluding parents who did not complete the 1-year follow-up test.

We explored whether there were differences in the effects of the podcast on parents depending on whether they had a primary, secondary or tertiary education level. We also explored whether there were differences in the effects of the podcast on parents who had a child in a school that received the IHC primary school resources and those whose children were in a control school. These analyses were adjusted for whether the child was in an intervention school and the parent’s level of formal education, respectively, which were our stratification variables at randomisation. Odds ratios from the logistic regression analyses were converted to risk differences using the control group odds as the reference, multiplying that by the odds ratio to estimate the intervention group odds, and converting the control and intervention group odds to probabilities to calculate the difference.

We calculated the adjusted standardised mean difference (Hedges’ *g*) [56] for comparison to effect sizes reported in a meta-analysis of the effectiveness of other interventions to improve critical thinking [57]. The statistical analyses were performed using R (R Core Team, Vienna, Austria; version 3.4.3; using packages MASS, tidyverse, compute.es, knitr, kableExtra, scales, and digest).

### Patient and public involvement

We constituted an advisory panel comprising members of the public, who advised on the design of the intervention (the IHC podcast). We worked with members of the public to refine prototypes of the podcast through iterative processes of human-centred design. Members of the public contributed the ideas for drama skits, the presentation and episode stories, explanations and examples, among others. We conducted user tests using feedback provided by members of the public, which we used to improve the podcast. Some participants helped in the recruitment when they invited their colleagues to recruitment meetings. The results will be shared with and explained to the parents.

## Results

**Fig. 1:**
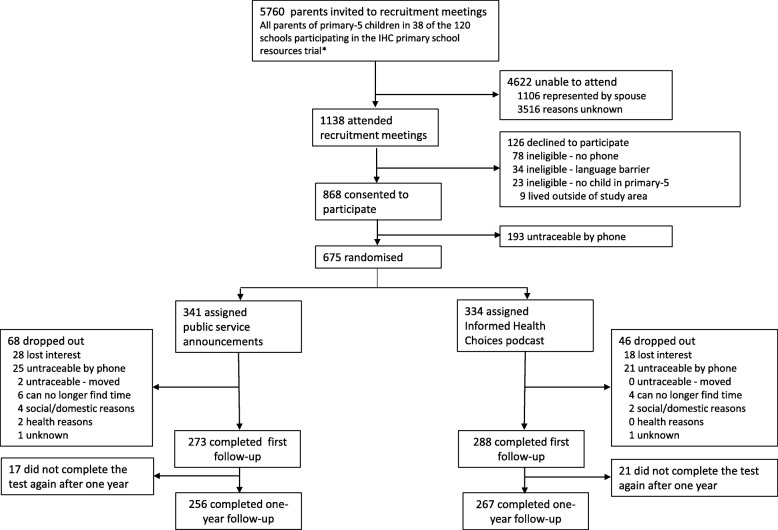
Informed Health Choices (IHC) podcast trial profile

Out of 675 parents who agreed to participate and could be reached by phone, 334 were randomly allocated to listen to the podcast and 341 were allocated to the public service announcements (control) group (Fig. 1). In the podcast group, 288 parents (86.2%) completed the test initially and 267 parents (80%) completed the test again after 1 year. In the control group, 273 (80.1%) completed the test initially and 256 parents (75%) completed the test again after 1 year. The education, sex, sources of health care and sources of advice about treatments were similar for parents in the podcast and control groups initially and on the 1-year follow-up (Table 5).

**Table 5:** Characteristics of the participants

	Control group (*n* = 341)^a^		Podcast group (*n* = 334)^a^	
	One-year follow-up	Initially after listening to the podcast^g^	One-year follow-up	Initially after listening to the podcast^g^
Completed tests	256 (75%)	273 (80%)	267 (80%)	288 (86%)
Education				
Primary	112 (44%)	144 (53%)	123 (46%)	145 (50%)
Secondary	68 (27%)	68 (25%)	85 (32%)	89 (31%)
Tertiary	74 (29%)	61 (22%)	58 (22%)	54 (19%)
Training in research^b^	130 (51%)	84 (31%)	147 (55%)	96 (33%)
Prior participation in research^c^	154 (60%)	74 (27%)	94 (35%)	72 (25%)
Sex				
Female	194 (76%)	208 (76%)	201 (75%)	221 (77%)
Male	62 (24%)	65 (24%)	66 (25%)	67 (23%)
Age				
Median (25th to 75th percentile)	38 (32 to 45)	37 (30 to 44)	36 (31 to 43)	35 (30 to 42)
(Range)	(19 to 74)	(18 to 74)	(19 to 79)	(18 to 77)
Sources of healthcare^d^				
Government health facility	177 (69%)	163 (60%)	192 (72%)	177 (61%)
Private not-for-profit health facility	20 (8%)	25 (9%)	27 (10%)	32 (11%)
Private for-profit health facility	82 (32%)	107 (39%)	84 (31%)	93 (32%)
Alternative medicine practitioners	5 (2%)	7 (3%)	9 (3%)	8 (3%)
Advice about treatments^e^				
Friends/relatives	25 (10%)	77 (28%)	20 (7%)	46 (16%)
Health workers	222 (87%)	183 (67%)	248 (93%)	236 (82%)
Community leaders	6 (2%)	4 (1%)	3 (1%)	6 (2%)
Radio/TV programmes	24 (9%)	31 (11%)	22 (8%)	19 (7%)
Alternative medicine practitioners^f^	4 (2%)	5 (2%)	5 (2%)	8 (3%)
Internet	3 (1%)	2 (1%)	8 (3%)	3 (1%)

^a^ Randomly allocated

^b^ “Have you ever had any training in scientific research (statistics, epidemiology or randomised trials)?”

^c^ “Have you ever been a participant in a scientific research study?”

^d^ “If you or your family member are unwell, where do you commonly seek medical attention?” (select all that apply)

^e^ “If you need to make a decision on what treatments to use, where do you usually get advice?” (select all that apply)

^f^ For example, herbal medicine practitioners

^g^Results of the initial evaluation were published elsewhere [43]

After 1 year, more parents responded that they had training in research in both the podcast and control groups. There was a larger increase in the number of parents who reported prior participation in research in the control group (from 27% to 60%) than in the podcast group (from 25% to 35%). This likely reflects participation in this study, and possibly a difference in whether they perceived their participation in this study as participation in research.

Nearly half the parents had no more than primary school education. About three quarters were women. The median age was 36 years (25th to 75th percentile, 31–43) in the podcast group and 38 years (25th to 75th percentile, 32–45) in the control group. The participants reported most commonly seeking health care at government or private for-profit facilities and they were most likely to seek advice about treatments from health workers.

### Primary outcomes and sensitivity analyses

**Fig. 2:**
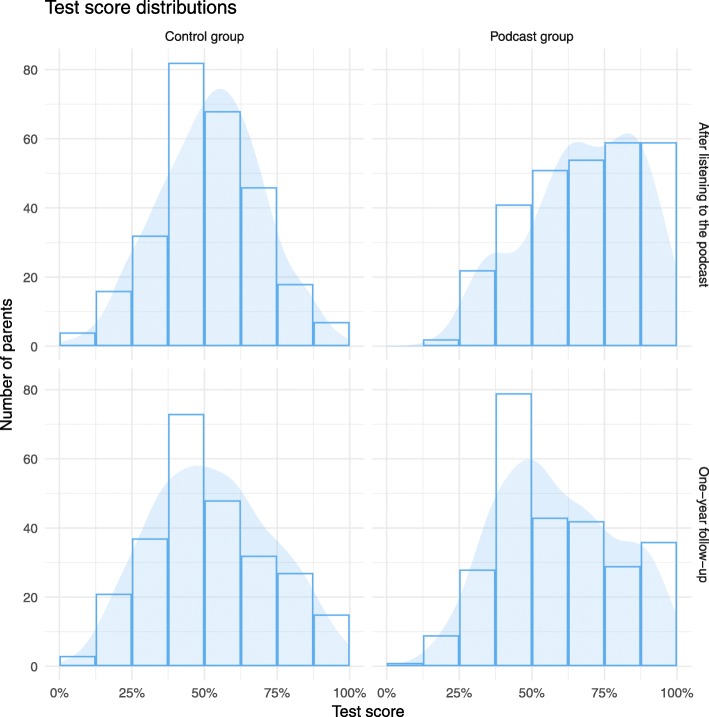
Test score distributions. Distribution of participants’ test scores from the test performed immediately after the intervention and that performed 1 year later

After 1 year, the mean score for parents in the podcast group went down from 67.8% initially after listening to the podcast to 58.9%, whereas there was little change in the control group, which was 52.6% after 1 year (up from 52.4%) (Table 6 and Fig. 2). The adjusted difference in mean scores between the podcast and control groups was 6.7% (95% CI 3.3% to 10.1%; *p* = 0.0001) after 1 year, compared to 15.5% after listening to the podcast initially.

**Table 6:** Main results

	Control group	Podcast group	Adjusted odds ratio^a^	Adjusted difference^a^
Primary outcome				
1 year after listening to the podcast Mean score, %	Mean score 52.6% (SD 20.4%)	Mean score 58.9% (SD 20.6%)		Mean difference: 6.7% (95% CI 3.3% to 10.1%) *p* = 0.0001
Initially after listening to the podcast Mean score (%)	Mean score 52.4% (SD 17.6%)	Mean score 67.8% (SD 19.6%)		Mean difference: 15.5% (95% CI 12.5% to 18.6%) *p* < 0.0001
1 year after listening to the podcast Passing score^b^	39.5% of parents (*n* = 101/ 256)	47.2% of parents (*n* = 126/267)	1.5 (95% CI 1.0 to 2.2) *p* = 0.03	9.8% more parents (95% CI 0.9% to 18.9%)
Initially after listening to the podcast Passing score (indicating a basic understanding of the key concepts)^b^	37.7% of parents (*n* = 103/ 273)	70.5% of parents (*n* = 203/288)	4.2 (95% CI 2.9 to 6.0) *p* < 0.0001	34.0% more parents (95% CI 26.2% to 40.7%)
Secondary outcomes				
1 year after listening to the podcast Mastery score^c^	10.5% of parents (*n* = 27/256)	19.5% of parents (*n* = 52/267)	2.2 (95% CI 1.3 to 3.7) *p* = 0.003	9.8% more parents (95% CI 2.8% to 19.6%)
Initially after listening to the podcast Mastery score^c^	6.2% of parents (*n* = 17/273)	31.6% of parents (*n* = 91/288)	7.2 (95% CI 4.1 to 12.4) *p* < 0.0001	26.0% more parents (95% CI 15.2% to 38.8%)

^a^ Odds ratios are adjusted for the stratification variables (education and child’s study group in the Informed Health Choices primary school trial). The odds ratios have been converted to differences using the control group as the reference

^b^ 11 or more correct answers out of 18 questions

^c^ 15 or more correct answers out of 18 questions

In the podcast group, 47.2% of the parents had a pass score after 1 year (down from 70.5%), compared to 39.5% in the control group (up from 37.7%) (Table 6). The adjusted difference (based on the odds ratio from the logistic regression analysis) was 9.8% more parents who passed (95% CI 0.9% to 18.9%; *p* = 0.03) in the podcast group than in the control group (compared to 34.0% more parents initially).

We conducted two sensitivity analyses to assess the potential risk of bias from attrition - parents who did not take the 1-year follow-up test. First, we calculated Lee’s treatment effect bounds for the mean difference in test scores. This resulted in a lower (worst case) and upper (best case) mean difference of 6.2% and 6.7%, respectively (95% CI 1.8% to 9.3%) (Table 7). This indicates that in the worst-case scenario, parents who listen to the podcast would be expected to score at least 6.2% higher on the test compared to parents who listen to typical public service announcements about health issues, and that this difference is statistically significant. Second, we calculated the adjusted mean difference and the adjusted difference in the proportion of parents with a passing score shortly after listening to the podcast (initial test), excluding participants lost to 1-year follow up. There was little difference between these analyses and the primary analyses, again indicating that there was little bias from attrition.

**Table 7:** Sensitivity analyses

	Control group	Podcast group	Adjusted odds ratio^a^	Adjusted difference^a^
One year after listening to the podcast				
Mean score				
Primary analysis	Mean score 52.6% (SD 20.4%)	Mean score 58.9% (SD 20.6%)		Mean difference: 6.7% (95% CI 3.3% to 10.1%) *p* < 0.0001
Lee bounds				6.2% to 6.7% (95% CI 1.8% to 9.3%)
Initially after listening to the podcast				
Mean score				
Primary analysis	Mean score 52.4% (SD 17.6%)	Mean score 67.8% (SD 19.6%)		Mean difference: 15.5% (95% CI 12.5% to 18.6%)
Excluding participants lost to 1-year follow up	Mean score 53.0% (SD 17.9%)	Mean score 67.6% (SD 19.7%)		Mean difference: 14.9% (95% CI 11.7% to 18.1%)
Passing score				
Primary analysis	37.7% of parents (*n* = 103/ 273)	70.5% of parents (*n* = 203/288)	4.2 (95% CI 2.9 to 6.0) *p* < 0.0001	34.0% more parents (95% CI 26.2% to 40.7%)
Excluding participants lost to 1-year follow-up	39.6% of parents (*n* = 101/ 255)	69.8% of parents (*n* = 187/268)	3.8 (95% CI 2.6 to 5.5) *p* < 0.0001	31.5% more parents (95% CI 23.4% to 38.6%)

^a^ Adjusted for the stratification variables (education and child’s study group in the Informed Health Choices primary school trial). The odds ratios from the logistic regressions for passing scores have been converted to differences based on the intervention school proportions and the odds ratios calculated using the intervention schools as the reference (the inverse of the odds ratios shown here)

### Secondary outcomes

Skills retention: there was a 29% relative reduction in the average ability of the parents in the podcast group over the year after listening to the podcast (71% retention, adjusted for chance) (Table 8). The relative reduction in the proportion of parents with a passing score was 33% (67% retention). For comparison purposes, we present the results of the parents together with those from a sister trial involving their children.

**Table 8:** Skill retention of parents and children

Outcomes^a^		Children^b^			Parents^b^		
	Follow up	Control	Intervention^c^	Retention in the intervention group^d^	Control	Intervention^c^	Retention in the intervention group^d^
Mean score	Initially	43%	63%	127%	52%	68%	71%
		Difference: 20% higher (95% CI 17% to 23% higher)			Difference: 16% higher (95% CI 13% to 19% higher)		
	After 1 year	53%	69%		53%	59%	
		Difference: 17% higher (95% CI 14% to 20% higher)			Difference: 7% higher (95% CI 3% to 10% higher)		
Passing score	Initially	27 per 100	69 per 100	116%	38 per 100	71 per 100	67%
		Difference: 50 more per 100 (95% CI 44 to 55 more)			Difference: 34 more per 100 (95% CI 26 to 41 more)		
	After 1 year	52 per 100	80 per 100		40 per 100	47 per 100	
		Difference: 40 more per 100 (95% CI 30 to 48 more)			Difference: 10 more per 100 (95% CI 1 to 19 more)		
Mastery score	Initially	1 per 100	19 per 100	155%	6 per 100	32 per 100	62%
		Difference: 18 more per 100 (95% CI 18 to 18 more)			Difference: 26 more per 100 (95% CI 15 to 39 more)		
	After 1 year	5 per 100	30 per 100		11 per 100	20 per 100	
		Difference: 25 more per 100 (95% CI 23 to 27 more)			Difference: 10 more per 100 (95% CI 3 to 20 more)		

^a^ A passing score for the children was 13 or more correct answers out of 24 questions, and a mastery score was 20 or more correct answers out of 24 questions. A passing score for the parents was11 or more correct answers out of 18 questions, and a mastery score was 15 or more correct answers out of 18 questions

^b^10,183 children completed the first test at the end of the term when the lessons were taught in the Informed Health Choices (IHC) primary school trial [44], and 6787 completed the second test after 1 year [45]. There were 561 parents who completed the first test in the IHC podcast trial after listening to the podcast and 523 completed the second test after 1 year

^c^The intervention in the IHC primary school trial included a workshop for the teachers, a textbook, exercise book, teacher’s guide, and nine 80-min lessons with reading, exercises and classroom activities. The differences are adjusted for stratification variables in both studies. The differences for the passing and mastery scores are based on the adjusted odds ratios, using the control groups as the reference

^d^The test scores in the intervention group after 1 year relative to the test scores shortly after the intervention in the intervention group. Retention for the mean score is adjusted for chance. There was a probability of the children answering 39% of the questions correctly by chance and of the parents answering 37% of the questions correctly by chance

In the podcast group, 19.5% of the parents had a score indicating mastery of the nine IHC key concepts after 1 year (down from 31.6%) compared to 10.5% of the parents in the control group (up from 6.2%). The adjusted difference was 9.8% more parents with a mastery score (95% CI 2.8% to 19.6%; *p* = 0.003) in the podcast group than in the control group (compared to 26.0% initially).

The proportion of parents who answered both questions correctly for each IHC key concept addressed in the podcast was higher in the podcast group than in the control group for eight of the nine key concepts (Additional file 2: Table S1). However, the differences were small for seven of those key concepts (3.3% to 9.4%; *p* 0.03–0.43) compared to the initial results. There was no clear difference for the key concept that treatments have both beneficial and harmful effects (adjusted difference 0.0%; 95% CI − 8.4% to 9.0%; *p* = 0.99); whereas for the closely related key concept that treatments can harm, 19.5% more participants in the podcast group answered both questions correctly (95% CI 10.4% to 28.6%; *p* < 0.0001). In contrast, the proportion of parents who answered both questions correctly for each key concept addressed in the podcast were between 13% and 35% higher for all nine concepts initially.

We detected no clear difference after 1 year between the podcast and control groups in how likely they would be to find out the basis for a claim about treatment effects or to find out if the claim was based on research (Additional file 2: Table S2). Parents in the podcast group were 12.6% less likely than parents in the control group to agree to participate in research about an illness they might get (95% CI − 22.3% to − 4.8%; *p* = 0.0005), whereas there was little if any difference initially. Most parents in both groups (65–86%) responded positively to all three of these questions.

Initially, parents in the podcast group were more likely than parents in the control group to respond that they found it easy or very easy to assess whether a treatment claim is based on research; to find research-based information about treatments; to assess how confident they could be about research results; and to assess the relevance of research. After 1 year, the proportion of parents in the podcast group who found these tasks to be easy or very easy decreased and there was no clear difference between the podcast and control groups (Additional file 2: Table S3).

There was little difference in how frequently parents in the podcast and control groups heard treatment claims (Additional file 2: Table S4). In the podcast group 62.2% of the parents reported hearing one or more claims most days or most weeks compared to 55.5% in the control group (adjusted difference 7.6% more in the podcast group; 95% CI − 1.0% to 15.4%; *p* = 0.08). The proportion of parents who responded that they thought about the basis for the last claim they heard was lower in the podcast group than in the control group (adjusted difference 8.2% less in the podcast group; 95% CI − 17.3% to 0.0%; *p* = 0.05) (Additional file 2: Table S5). However, parents in the podcast group were less likely to be very sure or not to know how to assess how sure they should be (adjusted difference 20.9% less in the podcast group; 95% CI − 29.9% to − 2.0%; *p* < 0.0001) (Additional file 2: Table S6). Parents in the podcast group were also less likely to be very sure about the advantages and disadvantages of the most recent treatment they used (adjusted difference 13.3% less in the podcast group; 95% CI − 19.9% to − 5.5%; *p* = 0.001) (Additional file 2: Table S7).

There was no clear difference in the proportion of parents whose assessment of the trustworthiness of the last claim they heard was consistent with what they identified as the basis for the claim (adjusted difference 3.8% more in the podcast group; 95% CI − 2.8% to 12.3%; *p* = 0.30). There was also little if any difference in the proportion of parents who responded that they were not sure because they did not know about the disadvantages.

The standardised mean difference (Hedges’ *g*) was 0.32 (95% CI 0.15 to 0.50). None of the parents or research assistants who delivered the podcasts reported any adverse effects.

### Subgroup analyses

The podcast was effective across parents with different levels of education (Additional file 2: Table S8). However, there was an interaction between parental education and the size of the podcast’s effect. The effect was largest for parents with tertiary education and lowest for parents with secondary education. There also was an interaction between having a child in a school that used the IHC primary school resources and the size of the effect (Additional file 2: Table S9). The effect of the podcast was less in parents who had a child in an intervention school. Neither of these interactions were consistent with what we had hypothesised, and we did not detect interactions for either of these factors in the initial results [44].

Overall, the mean score (percentage of correct answers) for parents with a child in an intervention school was 4.2% higher than that for parents with a child in a control school (95% CI 0.7% to 7.7; *p* = 0.02) and 11.9% more parents had a passing score (95% CI 2.8% to 21.2%; *p* = 0.01) after 1 year (Additional file 2: Table S10). This is in contrast to the initial results, when we did not find an association between having a child in a school that used the primary school resources and parents’ test scores.

## Discussion

The size of the effect of the IHC podcast decreased substantially over 1 year, largely because the parents did not retain what they had learned. In contrast, the children who were in schools that used the IHC primary school resources retained what they learned [44]. Moreover, after 1 year, parents who listened to the podcast were less likely than parents in the control group to have thought about the basis for the last claim that they heard and less likely to agree to participate in research; and their subjective ability to assess the trustworthiness of claims had decreased. On the other hand, they were less likely to be very sure or not to know how to assess how sure they should be about the last treatment claim that they heard.

There are several possible explanations for these findings. The decrease in scores in the podcast group might be due to the parents not regularly using what they had learned 1 year previously. Results of other studies on skill retention and skill decay have identified substantial skill loss with non-practice [58, 59].

There was a 33% relative decrease in the proportion of parents who had a passing score compared to a 16% relative increase for the children in intervention schools in the IHC primary school trial [44]. Differences between the interventions and differences between adults and children might explain this difference in retention.

We expected a larger effect for the children, because the primary school intervention was multifaceted, actively engaged the children and involved more time (about 12 h over 10 to 12 weeks compared to about 1.5 h over 7–10 weeks). Active, collaborative learning is generally more effective than passive learning and may improve retention [60]. Spaced practice, with intervals between learning sessions, has been found to improve long-term retention [59, 61]. Listening to the podcast did not include any practice, other than encouraging the parents to think carefully when they hear a claim. Learners need immediate practice to move information from working memory to long-term memory [62]. Just seeing or hearing new concepts may not be enough for learning. The mind has to do some work with new information before it is reliably stored in memory.

Another potentially important difference between the podcast and the primary school interventions is that parents listened to the podcast alone. People learn from one another [63]. The research assistants who delivered the podcast episodes did not discuss the podcast with the parents. Although some parents shared the podcast with others [47], we did not actively encourage discussion of the podcast. In contrast, the primary school intervention took place in classrooms with discussion, modelling and opportunities for observation and imitation. Also, teachers were able to make adjustments to help ensure that the children’s understanding, by asking questions, using additional examples, providing additional explanations, working through activities and reviewing exercises together.

In addition to differences between the interventions, there are differences in learning between children and adults. Children are expected to be in school learning, whereas adults have other responsibilities. Adults are also more likely to have well-established routines, they may expect learning to come effortlessly, and they may be less able than children to learn cognitive skills [62]. It can take time and many demonstrations to convince adult learners of the superiority of new routines over old-established ones. They have had their misconceptions for longer than children, and may not recognise them or see them as dysfunctional. For example, some parents who participated in the process evaluation had strong prior beliefs and remained steadfast in those beliefs after listening to the podcast, even when those beliefs were in conflict with a podcast message [46].

Adults may expect learning to come effortlessly, forgetting how they worked as children to learn new concepts. When new cognitive skills are learned, it may take a lot of thought and effort, because initially they are stored in declarative (factual) rather than procedural memory [63]. Some aspects of memory, reasoning, problem solving and intellectual tasks may begin to deteriorate in the 30’s age group [64, 65]. The median age of the participants in the podcast group was 35 years (25th to 75th percentile, 31–43).

The findings for each key concept were largely consistent with the overall results and what we found initially after listening to the podcast. The scores decreased for all of the concepts. Both the initial test and the test after 1 year showed the largest effect for the concept that treatments may be harmful, and the smallest effect (no clear effect in this study) for the concept that treatments usually have beneficial and harmful effects. These two concepts are closely related, but these findings support considering them separately, and suggest that the first may be more of a problem than the second. People often exaggerate the benefits of treatments, ignore or downplay potential harms, overestimate the benefits and underestimate harm [23]. On the other hand, people are generally aware that it is important to consider the balance between benefits and harms when making a decision. It is also possible that the difference we found between these two key concepts was influenced by the nature and difficulty of the questions that were used.

We know from the process evaluation that at least some parents shared the IHC podcast with neighbours [46], but we do not know the extent to which parents in the podcast group shared the podcast with parents in the control group. Nonetheless, given that there was little change in the scores of the control group from the first to the second test, if there was contamination, it is unlikely to have had a substantial effect on the scores of the parents in the control group after 1 year.

So far as we are aware, this is the first randomised trial of the use of a podcast for non-formal education or health education, other than a podcast to aid weight loss [6, 7, 27, 37–49]. Few other interventions to improve the ability of non-health-professionals to think critically about treatments have been evaluated [35, 36]. A systematic review of strategies to teach people to think critically more broadly, which included 308 studies, found an average effect size (Hedges’ *g*) of 0.33 [57]. The average effect size for interventions that were targeted at graduate and adult students was 0.21, as was the average effect size for interventions in health or medical education. The effect size for our intervention shortly after listening to the podcast (0.83) was large in comparison. The effect size after 1 year was 0.32, which is closer to the average effect size for interventions targeted at adults. However, comparisons such as these must be made cautiously due to differences in the interventions compared in these studies, the outcome measures and the methods that were used.

### Strengths

It is unlikely that the main findings of this study can be explained by random errors. We also believe there is a low risk of systematic errors (bias). The comparison groups were similar at the start of the study, they were managed similarly apart from the intervention, and outcomes were measured in the same way in both groups. There was more loss to follow up in the control group than in the podcast group (25% versus 20%), but there were no clear differences between those who completed the tests and those who dropped out. Although loss to follow up affected the precision of our estimates and may have introduced some bias, it seems unlikely to have had an important impact on the main findings of the study.

### Limitations

The applicability of our findings is limited by the nature of the intervention and the outcome measure that we used. The podcast was tailored to a specific target audience - parents of primary school children in Uganda. For a podcast to be effective in another audience, it would need to be tailored to that audience [66]. Although we were careful to ensure the reliability and validity of our primary outcome measure, it was designed to measure the ability to apply the concepts that the podcast was designed to teach (“treatment inherent”). Treatment-inherent outcome measures are associated with larger effect sizes than independent measures [57, 67]. In addition, we cannot be certain about the extent to which this outcome reflects how people apply the IHC key concepts when they hear health claims in their daily lives. Our findings on actual claim assessment and decision-making behaviours are based on self-report, are inconsistent and may be unreliable. Furthermore, the parents in the podcast trial volunteered to participate. Consequently, the effect estimates from this trial indicate the potential effects of the podcast amongst parents who choose to listen to them, not the effect of simply offering the podcast to a group of parents.

### Implications of these findings

Currently, many interventions for equipping people with the skills to think more critically about treatments are focused on health profession students, health workers and researchers. Overall, findings from our initial study suggest that developing mass media programmes for improving people’s ability to think more critically about treatments could be a beneficial investment. However, as demonstrated by the decay shown in the current study, in order for this investment to yield sustainable learning outcomes, such interventions need not be one-off and perhaps not be passive. Our assessment is that passive dissemination of media interventions is unlikely to be as effective as what we found our intervention to be initially after the intervention was delivered and certainly not effective a year later. Future research could include developing a spiral curriculum for teaching the IHC key concepts to adults, how to engage stakeholders to support teaching critical thinking about treatments to adults, developing outcome measures for research on making decisions about treatments, and systematic reviews of outcome assessment tools, frameworks and teaching strategies for critical thinking about treatments, among others.

## Conclusions

Critical health literacy is essential for informed health choices. Yet, despite worldwide recognition of the need to improve health literacy, up to now there have been only a handful of evaluations of interventions to improve health literacy in community populations [43]. We have shown that it is possible for adults in a low-income country, mostly with no more than primary school education, to improve their short-term ability to assess claims about treatment effects by listening to a podcast. However, more active, collaborative learning strategies with spaced practice are likely to be needed to address the substantial decay that we found in these skills and in self-efficacy over 1 year. In contrast to this decay in skills, we found an increase in the same skills among children in the intervention group of the IHC primary school trial [44]. Taken together, these findings provide further support for the importance of beginning to teach these skills at a young age.

## Supplementary information

**Additional file 1.** The Claim Evaluation Tools.

**Additional file 2: Table S1.** Results for each concept one year after listening to the podcast. **Table S2.** Intended behaviours. **Table S3.** Self-efficacy. **Table S4.** Self-reported behaviour - awareness of treatment claims. **Table S5.** Self-reported behaviour - assessment of the basis of treatment claims. **Table S6.** Self-reported behaviour - assessment of trustworthiness of treatment claims. **Table S7.** Self-reported behaviour - assessment of advantages and disadvantages of treatments. **Table S8.** Subgroup analyses - education. **Table S9.** Subgroup analyses - child in school that used IHC primary school resources. **Table S10.** Effect of IHC primary school resources on parents.

## Data Availability

The data files for the 1-year follow up are available from the Norwegian Centre for Research Data (http://www.nsd.uib.no/nsd/english/index.html).
