# Supplementary material for: Effects of the Informed Health Choices podcast on the ability of parents of primary school children in Uganda to assess the trustworthiness of claims about treatment effects: one-year follow up of a randomised trial
Source: Trials. 2020 Feb 14;21:187. doi: 10.1186/s13063-020-4093-x (PMC7023790; doi:10.1186/s13063-020-4093-x)
Supplement: Supplementary file 1 — Additional file 1. The Claim Evaluation Tools. [file 13063_2020_4093_MOESM1_ESM.pdf]

The Claim Evaluation Tools

This questionnaire includes multiple-choice questions about treatment claims. Please answer all questions to the best of your ability.

The questionnaire includes some words that may be unfamiliar to you:

A **TREATMENT** is anything done to care for yourself, so you stay well or, if you are sick or injured, so you get better and not worse. For example, wearing glasses (to see better).
IN LUGANDA: OBUJJANJABI

A **TREATMENT CLAIM** is something someone says about whether a treatment causes something to happen or to change. A claim can be true or can be false. For example, that wearing glasses makes you see better.
IN LUGANDA: EKINTU EKYOGERWAYOGERWA KUBY'OBUJJANJABI

A **RESEARCH STUDY** is a way to answer a question by carefully collecting information. For example, a study might be done to answer the question: Does wearing glasses make people see better?
IN LUGANDA: OKUNOONYEREZA OKWEKINNASAYANSI

RESULTS of a study are what the study found. For example, whether people who wear glasses could see better.
IN LUGANDA: EKIZUULIBWA OKUVA MUKUNOONYEREZA

Part 1. Questions about you

1.1 What is your name? ____________________________

1. 2 How old are you? _________

1.3 What is your gender?

☐ Female

☐ Male

1.4 Have you ever had any training in scientific research (statistics, epidemiology, randomised controlled trials)?

☐ Yes

☐ No

1.5 Have you ever been a participant in a scientific research study?

☐ Yes

☐ No

1.6 What level of education have you completed?

☐ No education

☐ Primary education

☐ Secondary education

☐ Tertiary education/ university

Part 2. Questions about claims

Instructions: Read the text above each question and then answer the question using one of the provided answers. For each question, choose what you think is the best answer and write the letter for that answer in the box provided.

Example

A teacher says that the children in his school run faster than the children going to school in another village.

Question: **How can the teacher be sure about this?**

Options:

A) He should ask a teacher at the other school

B) He should arrange for a running contest between the two schools

C) He should ask the children in his school what they think

D) He should ask the children in the other school what they think

Answer: B

Start

2. A doctor did a research study to find out if drinking tea keeps people from getting sick. He tossed a coin to decide who should get the tea and who should not. People who got tea went to the doctor's office every day to drink their tea. At the end of the study, people who got the tea were less likely to be sick than those who got no tea.

Based on the text above, please answer the following questions:

2.1 Who went to the doctor's office every day?

Options:

A) People who did not get tea

B) People who got tea

C) Everyone

D) People who got sick

Answer: ☐

2.2 How did the doctor decide who should get tea?

Options:

A) By tossing a coin

B) By asking people what they would like

C) He gave tea to those who were more likely to be sick

D) He asked people who came to his office

Answer: ☐

3. A doctor did a research study to find out if drinking tea keeps people from getting sick. He tossed a coin to decide who should get the tea and who should not. People who got tea went to the doctor's office every day to drink their tea. At the end of the study, people who got the tea were less likely to be sick than those who got no tea.

Based on the text above, please answer the following questions:

3.1 What was the treatment?

Options:

A) Tea

B) Sleep

C) The study

D) The doctor

Answer: ☐

3.2 What was the result of the study?

Options:

A) Drinking tea can help people from getting sick

B) Doctors should toss coins when doing studies

C) People should go to the doctor if they are sick

D) Not drinking tea can help people from getting sick

Answer: ☐

4. Annette sees an advert on TV for a new soap which the makers say protects people from getting skin rashes. Annette thinks that this soap must be better than other soaps for protecting her skin.

Question: **Is Annette right?**

Options:

A) No, the soap may be newer, but that does not mean that it is better than other soaps

B) Yes, the new soap is probably better than most other soaps because it is newer

C) Yes, the new soap is probably better than most other soaps because a well-known company makes it

Answer: ☐

5. Regina has an illness that makes it difficult for her to breathe. She hears on the radio about a medicine that has helped many people for their breathing problems.

Question: **How sure can Regina be that the medicine does not have any harms?**

Options:

A) It is not possible to say. However, medicines are rarely harmful

B) Not very sure, because all medicines may harm people as well as help them

C) Very sure, since the medicine has helped many people, it is unlikely that it also harms people

Answer: ☐

6. John has a skin rash on his leg. A shop sells several creams to treat skin rashes. John chooses a cream from a well-known company, even though it is more expensive than the other creams. John thinks the cream is more likely to heal his rash than the other creams because it is more expensive.

Question: **Is John right?**

Options:

A) No, just because the cream is expensive does not mean that it will work better than other creams

B) It is not possible to say. However, expensive creams are likely to be better because the companies spend more time making them

C) No, the cream is probably not as good as the other creams. Well-known companies are usually better at advertising

D) Yes, the company is well-known for a reason, so it is more likely to be better than creams sold by lesser-known companies

Answer: ☐

7. Two companies make two different medicines for treating stomach pain. Each of them says that their medicine is the better one.

Question: **How can you know which of the two medicines is better for stomach pain?**

Options:

A) It is not possible to say. The companies may just say their medicine is best because they want to make money

B) I would rely on the best known company; it is more likely to have the best medicine

C) I cannot trust either of the companies. They are probably both wrong

Answer: ☐

8. Dr. Kato and Dr. Semakula disagree about which medicine for stomach pain is best. Dr. Kato says his opinion is right because he has worked as a doctor for a longer time than Dr. Semakula.

Question: **Is Dr. Kato right?**

Options:

A) Yes, because Dr. Kato has worked for a long time, he has more experience than Dr. Semakula

B) Yes, because Dr. Kato has worked for a long time, he must be basing what he says on studies comparing the medicines

C) No, just because Dr. Kato has worked as a doctor for a longer time does not mean that he is basing what he says on studies that compare medicines for stomach pain

Answer: ☐

9. Habibah has pain in her ear, and she asks her brother Hassan what to do about it. He says that once, when he had a pain like that, he rinsed his ear with hot water. The next day, his ear pain was gone. Based on his experience, he says rinsing with hot water is helpful for ear pain.

Question: **Do you agree with Hassan?**

Options:

A) Yes. Because this is Hassan's experience, it is likely to be true

B) No, Hassan's experience is not enough to be sure

C) Yes, Hassan rinsed his ear with hot water and the next day his ear pain was gone

Answer: ☐

10. Sarah has an illness. There is a medicine for it, but she is unsure if she should try it. A research study comparing the medicine with no medicine found that the medicine was helpful but also that it could be harmful. Three of Sarah's friends are giving her advice about what to do.

Question: **Which advice below given to her by her friends is the best advice?**

Options:

A) She should only take the medicine if many people have tried the medicine before

B) She should only take the medicine if she thinks it will help her more than it will harm her

C) If Sarah has enough money to buy the medicine, it could not hurt to try it

Answer: ☐

11. Dr. Acheng is an expert on treating headaches. A news reporter interviews Dr. Acheng about a new medicine. Dr. Acheng says that, in her personal experience, the new medicine is good for treating headaches.

Question: **How sure can we be that Dr. Acheng is right?**

Options:

A) It is not possible to say. It depends on how long Dr. Acheng has been an expert on treating headaches

B) Not very sure. Even though Dr. Acheng is an expert, the new medicine still needs to be compared in studies with other treatments

C) Very sure. Dr. Acheng is an expert, so she knows if the new medicine is good or not based on her experience

D) Very sure. Dr. Acheng would not be interviewed by a news reporter if her advice was not good

Answer: ☐

12. Edith has a stomach pain. Edith's mother says that fruit juice is a good treatment for stomach pain. She learnt about this treatment from Edith's grandmother. Over many years, other families she knows have also used fruit juice to treat stomach pain.

Question: **Based on this, how sure can we be that fruit juice is a good treatment for stomach pain?**

Options:

A) Not very sure. Even though people have used fruit juice over many years, that does not mean that it helps stomach pain

B) Very sure. If it has worked for Edith's mother and other people who have tried it, it will probably work for her too

C) Not very sure. Edith should ask more families if they use fruit juice to treat stomach pain

Answer: ☐

13. At David's school, some students have poor parents. The students with poor parents drink less fruit juice than the children of other parents. The students with poor parents are also more often sick. Based on this link, David thinks that people who drink fruit juice, are less likely to get sick.

Question: **Is David correct?**

Options:

A) It is not possible to say, it depends on whether or not David has poor parents

B) Yes, students with poor parents do not drink fruit juice and are more often sick

C) Yes, the juice is the only possible reason why the students with the poor parents are more often sick

D) It is not possible to say. There could be other reasons why students with poor parents are more often sick

Answer: ☐

14. In a research study done by John, four people were told to do exercises every day for a month, and four people were told to eat bananas every day. At the end of the month, the people who ate bananas had more strength than those who did exercises. Based on his study, John advises his friend Mildred to eat bananas.

Question: **Mildred says that we cannot be sure about the results of John's study. Why?**

Options:

A) Because the study included so few people, the differences in strength could have happened by chance, and not because of the bananas

B) Because John should have included fewer people in his study so that he could have followed them more closely

C) Because four people is not enough, all people taking part in the study should have been told to eat the bananas

Answer: ☐

15. A new fruit drink is said to make people feel strong. Fred wanted to know if this is true, and decided to do a research study comparing people who got the new fruit drink and people who drank just water.

People in the study knew if they got the new drink or water, and Fred told them that the new fruit drink was likely to make people stronger. At the end of the study, Fred was right and those who drank the new fruit drink said they felt stronger.

Question: **Why can't we be sure about the results of Fred's study?**

Options:

A) Because all people taking part in the study should have been given the new fruit drink

B) Because people knew if they got the new fruit drink, and knowing this may have influenced how they felt

C) Because Fred should have told both groups that they could expect to feel stronger

Answer: ☐

16. Harriet is worried about getting sick. She hears about a new research study on the radio that compared a new medicine to an old medicine. Fewer people who took the old medicine got sick compared to the people who took the new medicine.

Question: **How sure can Harriet be that the old medicine is better than the new medicine?**

Options:

A) Not so sure, because Harriet needs to know the results of all other studies comparing the new medicine with the old medicine

B) Very sure, because she heard about the study on the radio

C) Not so sure, unless she finds another study with the same results

D) Very sure, because this is a new study

Answer: ☐

17. Doctors studied people with stomach pain before and after they took a new medicine. After taking the new medicine, many people felt less pain.

Question: **Can we be sure that the new medicine is good for treating stomach pain?**

Options:

A) No, taking the new medicine should have been compared either with not taking the medicine, or with taking an older medicine

B) Yes, people were asked how much pain they felt before and after they took the new medicine

C) Yes, the study was done by doctors

Answer: ☐

18. In a research study, doctors compared two treatments for knee pain, a new treatment and an old treatment. People were able to choose which treatment they got. Most young people chose the new treatment. At the end of the study, people who chose the new treatment had less pain.

Question: **How sure can you be that the new treatment is better for treating pain than the old treatment?**

Options:

A) Not very sure, because people taking the new treatment and the old treatment were not similar

B) Not very sure, because all people taking part in the study should have got both treatments

C) Not very sure, because older people did not like the new treatment

Answer: ☐

19. Judith wants smoother skin. The younger girls in her school have smoother skin than the older girls. Judith thinks this is because the younger girls use cream on their skin to make the skin smoother.

Question: **Based on this link between using cream and smooth skin, is Judith correct?**

Options:

A) It is not possible to say. It depends on how many younger and older girls there are

B) It is not possible to say. There might be other differences between the younger and older girls

C) Yes, because the younger girls use cream on their skin and they have smoother skin

D) No, Judith should try using the cream herself to see if it works for her

Answer: ☐

20. Dr. Wasswa has done a research study giving a new medicine to people who were vomiting. Some of the people stopped vomiting after they got the new medicine. Dr. Wasswa says that this means that the medicine works.

Question: **Is Dr. Wasswa right?**

Options:

A) No. The people who used the medicine were not compared with similar people who did not use the medicine

B) Yes, some of the people stopped vomiting

C) No, since not all of the people stopped vomiting

Answer: ☐

Instructions: Read the text at the top of the box. Then read the text in each row and choose what you think is the best answer by making a tick ✓ in one of the two boxes. There should be only one tick in each row.

**21.** When you are sick, sometimes people say that something - a treatment - is good for you. Below you will find different things people say about such treatments.<br><br>**Do you agree or disagree with each of the following things being said?**		
For each thing being said below, use a tick ✓ to mark whether you "agree" or "disagree".		
**Things being said:**	**I agree**	**I disagree**
**21.1** Peter says that if a treatment works for one person, the treatment will help others too	☐	☐
**21.2** Alice says that if some people try the treatment and feel better, this means that the treatment helps	☐	☐
**21.3** Habibah says that, just because many people are using the treatment, this does not mean that it helps	☐	☐
**21.4** Julie says that companies sometimes say that the treatment they make is best just to make money	☐	☐

22. A doctor wanted to know if a new medicine for treating headaches is better than an older medicine. The doctor did a research study, comparing the two medicines.

Would the actions below make you more sure or less sure about the results of the study?

For each action below, use a tick ✓ to mark whether you think the action would help you become "more sure" or "less sure".

**Actions:**	**More sure**	**Less sure**
**22.1** The doctor should use chance (like tossing a coin) to decide which people should be given the new medicine and which should be given the old medicine	☐	☐
**22.2** People should not know which medicine they get (the new medicine or the old medicine) until the end of the study	☐	☐
**22.3** The doctor should include only a small number of people in the study	☐	☐

**23.** To know if a treatment helps you, the treatment should be compared in research studies to other treatments (fair comparisons). Below you will find different things people say about such studies.<br><br>**Do you agree or disagree with each of the following things being said?**
For each thing being said below, use a tick ✓ to mark whether you "agree" or "disagree".

**Things being said:**	**I agree**	**I disagree**
**23.1** Julie says that, if a treatment has been compared in a study to another treatment, you don't have to look for more studies	☐	☐
**23.2** Margaret says that the results of a study should be used to decide if a treatment is more helpful than harmful	☐	☐

Part 3. Questions about your views

Instructions: For the following questions, there are no right or wrong answers. Read the text at the top of the box. Then read the text in each row and <u>choose what you think is the best answer by making a tick ✓ in one of the five boxes</u>. There should be only one tick in each row.

**24.** Think about an illness that you might get. Imagine someone claiming (saying) that a particular treatment might help you get better.<br><br>**How likely are you to do each of the following actions?**					
(Mark with a tick ✓ in the box; one check for each row.)					
**Actions:**	**Very unlikely**	**Unlikely**	**Likely**	**Very likely**	**I don't know**
**24.1** Find out what the claim was based on (for example by asking the person making the claim)	☐	☐	☐	☐	☐
**24.2** Find out if the claim was based on a research study comparing the treatment to no treatment (a fair comparison)	☐	☐	☐	☐	☐

25. Below are some actions. Please read each one carefully and give the answer that comes closest to **how difficult or easy you find each of the actions to be:**

(Mark with a tick ✓ in the box; one check for each row.)

**Actions:**	**Very difficult**	**Difficult**	**Easy**	**Very easy**	**I don't know**
**25.1** Assessing whether a claim about a treatment is based on a research study comparing treatments (a fair comparison)	☐	☐	☐	☐	☐
**25.2** Assessing where I can find information about treatments that is based on research studies comparing treatments (fair comparisons)	☐	☐	☐	☐	☐
**25.3** Assessing how sure I can be about the results of a research study comparing treatments (the trustworthiness of the results)	☐	☐	☐	☐	☐
**25.4** Assessing if the results of a research study comparing treatments are likely to be relevant to me	☐	☐	☐	☐	☐

**26.** Think about an illness that you might get. **How likely are you to say “yes” if you are asked to participate in a research study comparing two treatments for your illness (a fair comparison)?**				
(Mark with a tick ✓ in one box)				
**Very unlikely**	**Unlikely**	**Likely**	**Very likely**	**I don’t know**
☐	☐	☐	☐	☐

Informed Healthcare Choices

Instructions: For the following questions, <u>choose what you think is the best answer and write the letter for that answer in the box provided.</u>

27. It is common for people to say that something will help improve your health or that it will not help. Some may say that it will be bad for your health. What people say about treatments may be correct but sometimes it may be wrong. We call these treatment claims.

How often do you hear treatment claims?

Options:

A) One treatment claim or more on most days

A) One treatment claim or more during most weeks

B) One treatment claim or more during most months

C) I almost never hear treatment claims

D) I don’t know

Answer: ☐

28.1 When was the last time you heard a treatment claim?

Options:

A) This week
B) Last week
C) Last month
D) More than a month ago
E) I have never heard of any treatment claims

Answer: ☐

28.2 A treatment is anything done to care for yourself, so you stay well or, if you are sick or injured, so you get better and not worse

What was the **treatment** in the claim you last heard about?

Options:

A) Using a medicine (for example, taking a tablet or a syrup)
B) Getting an operation (for example, removing a bad tooth)
C) Using something to feel better or to heal more quickly (for example, using a bandage, or glasses)
D) Eating food or drinking something to feel better (for example, herbs or fruit)
E) Avoiding doing something to feel better (for example, not drinking milk)
F) Something else

Answer: ☐

28.3 In the space below please write down the treatment claim that you last heard. (What did they say the treatment would change or not change about someone's health?)

____

____

____

____

____

28.4 Did you think about what that treatment claim that you heard was based on?

Options:

A) No, I did not think about what the treatment claim was based on

B) Yes, I thought about what the treatment claim was based on

C) I don't remember thinking about it

Answer: ☐

28.5 If you heard about a treatment claim, what was it based on?

It was based on:

- **A)** Someone's personal experience using the treatment
- **B)** What an expert said about it
- **C)** A research study that compared the treatment with another treatment or no treatment
- **D)** Something else
- **E)** I could not tell what the treatment claim was based on

Answer: ☐

28.6 How sure are you that the treatment claim you heard is true or can be trusted?

Options:

- **A)** Not very sure because I don't know the reason behind the claim
- **B)** Not very sure because the reason behind the claim was not good
- **C)** Very sure because the reason behind the claim was good
- **D)** I don't know because I don't know how to decide whether it is true or not

Answer: ☐

29.1 When was the last time you yourself decided whether to use a treatment or not to use a treatment?

Options:

A) This week

B) Last week

C) Last month

D) More than a month ago

E) I have never decided to use or not to use a treatment

Answer: ☐

29.2 When was the last time that someone else decided for you whether you should use a treatment or not use a treatment?

Options:

A) This week

B) Last week

C) Last month

D) More than a month ago

E) No one else has ever decided for me to use or not to use a treatment

F) I can't remember

Answer: ☐

29.3 What was the treatment for which you or someone else made the decision?

Options:

A) Using a medicine (for example, taking a tablet or a syrup)
B) Getting an operation (for example, removing a bad tooth)
C) Using something to feel better or to heal more quickly (for example, using a bandage, or glasses)
D) Eating food or drinking something to feel better (for example, herbs or fruit)
E) Avoiding doing something to feel better (for example, not drinking milk)
F) Something else

Answer: ☐

29.4 It is common for people to say that something will help improve your health or that it will not help. Some may say that it will be bad for your health. What people say may be correct but sometimes it may be wrong. We call these treatment claims.

What was the claim about the treatment for which you made the decision? (What did they say the treatment would change or not change about your health?)

____

____

____

29.5 Did you think about the possible reasons behind what they said about what the treatment would do or not do?

Options:

A) No. I did not think about the possible reasons behind what they said

B) Yes. I thought about the possible reasons behind what they said

C) I don't remember thinking about the possible reasons behind what they said

Answer: ☐

29.6 What were the reasons behind what they said the treatment would change about your health?

Options:

A) Someone's personal experience using the treatment

B) What an expert said about it

C) A research study that compared the treatment with another treatment or no treatment

D) Something else

E) I don't remember what the treatment claim was based on

Answer: ☐

29.7 What did you yourself decide to do about the treatment?

A) I did not use the treatment
B) I used the treatment
C) I waited because I wanted to know more about the treatment
D) Someone else decided for me

Answer: ☐

29.8 How sure are you about the advantages and disadvantages of the treatment you used?

Options:

A) Not very sure because I don't know the reasons behind the claims about the good and bad things that treatment makes happen

B) Not very sure because there was not a good reason behind the claims about the advantages of the treatment

C) Not very sure because I only know about the advantages of the treatment. I also need to know about the disadvantages

D) Very sure because there is a good reason behind the claims about the advantages and disadvantages of the treatment

E) I did not use any treatment

Answer: ☐

30. Who do you think should decide for you whether you should use a treatment or not use a treatment?

Options:

A) Me alone

B) My relative alone

C) Me and my relative

D) A person who knows a lot about treatments

E) Me and a person who knows a lot about treatments

F) Me, my relative and a person who knows a lot about treatments

Answer: ☐
