# Supplementary material for: Effects of the Informed Health Choices podcast on the ability of parents of primary school children in Uganda to assess the trustworthiness of claims about treatment effects: one-year follow up of a randomised trial
Source: Trials. 2020 Feb 14;21:187. doi: 10.1186/s13063-020-4093-x (PMC7023790; doi:10.1186/s13063-020-4093-x)
Supplement: Supplementary file 2 — Additional file 2: Table S1. Results for each concept one year after listening to the podcast. Table S2. Intended behaviours. Table S3. Self-efficacy. Table S4. Self-reported behaviour - awareness of treatment claims. Table S5. Self-reported behaviour - assessment of the basis of treatment claims. Table S6. Self-reported behaviour - assessment of trustworthiness of treatment claims. Table S7. Self-reported behaviour - assessment of advantages and disadvantages of treatments. Table S8. Subgroup analyses - education. Table S9. Subgroup analyses - child in school that used IHC primary school resources. Table S10. Effect of IHC primary school resources on parents. [file 13063_2020_4093_MOESM2_ESM.docx]

**Table S1. Results for each concept one year after listening to podcast**

| **Concept** | **Control**  **group**  % correct*  (n=256) | **Podcast group**  % correct*  (n=267) | **Adjusted odds ratio^†^**  (95% CI) | **Adjusted difference^†^**  (95% CI) |
| --- | --- | --- | --- | --- |
| **Claims** |  |  |  |  |
| Treatments may be harmful | 85 (33.2%) | 135 (50.6%) | 2.2  (95% CI 1.6 to 3.3)  p<0.0001 | 19.5%  (95% CI 10.4% to 28.6%) |
| Personal experiences or anecdotes (stories) are an unreliable basis for assessing the effects of most treatments | 105 (41.0%) | 125 (46.8%) | 1.3  (95% CI 0.9 to 1.8)  p=0.22 | 5.5%  (95% CI -3.1% to 14.4%) |
| An ‘outcome’ may be associated with a treatment, but not caused by the treatment | 89 (34.8%) | 108 (40.4%) | 1.4  (95% CI 0.9 to 2.0)  p=0.10 | 7.3%  (95% CI -1.3% to 16.4%) |
| Widely used treatments or treatments that have been used for a long time are not necessarily beneficial or safe | 85 (33.2%) | 101 (37.8%) | 1.2  (95% CI 0.9 to 1.8)  p=0.28 | 4.6%  (95% CI -3.5% to 13.4%) |
| New, brand-named, or more expensive treatments may not be better than available alternatives^‡^ | 128 (50.0%) | 109 (40.8%) | 0.7  (95% CI 0.5 to 1.0)  p=0.07 | -8.3%  (95% CI -16.6% to 0.5%) |
| Opinions of experts or authorities do not alone provide a reliable basis for deciding on the benefits and harms of treatments | 75 (29.3%) | 92 (34.5%) | 1.3  (95% CI 0.9 to 2.0)  p=0.13 | 6.3%  (95% CI -1.7% to 15.4%) |
| Conflicting interests may result in misleading claims about the effects of treatments^‡^ | 105 (41.0%) | 98 (36.7%) | 0.9  (95% CI 0.6 to 1.2)  p=0.43 | -3.5%  (95% CI -11.5% to 5.3%) |
| **Comparisons** |  |  |  |  |
| Identifying effects of treatments depends on making comparisons | 32 (12.5%) | 49 (18.4%) | 1.8  (95% CI 1.1 to 2.9)  p=0.03 | 7.5%  (95% CI 0.7% to 17.0%) |
| Apart from the treatments being compared, the comparison groups need to be similar at the beginning of a comparison (i.e. ‘like needs to be compared with like’) | 80 (31.2%) | 92 (34.5%) | 1.2  (95% CI 0.8 to 1.7)  p=0.43 | 3.3%  (95% CI -4.5% to 12.0%) |
| If possible, people should not know which of the treatments being compared they are receiving^‡^ | 81 (31.6%) | 85 (31.8%) | 1.1  (95% CI 0.7 to 1.5)  p=0.79 | 1.1%  (95% CI -6.5% to 9.8%) |
| Small studies in which few outcome events occur are usually not informative and the results may be misleading^‡^ | 95 (37.1%) | 119 (44.6%) | 1.4  (95% CI 1.0 to 2.1)  p=0.05 | 8.7%  (95% CI -0.1% to 17.8%) |
| The results of single comparisons of treatments can be misleading | 74 (28.9%) | 99 (37.1%) | 1.5  (95% CI 1.1 to 2.2)  p=0.03 | 9.4%  (95% CI 1.0% to 18.7%) |
| **Choices** |  |  |  |  |
| Decisions about treatments should not be based on considering only their benefits | 105 (41.0%) | 104 (39.0%) | 1.0  (95% CI 0.7 to 1.4)  p=0.99 | 0.0%  (95% CI -8.4% to 9.0%) |

* There were two multiple-choice questions for each concept. The proportions are for the percentage of children who answered both questions correctly.

^†^ The odds ratios are adjusted for the stratification variables (education and child’s study group in the Informed Health Choices primary school trial). The odds ratios have been converted to differences using the control group as the reference.

^‡^ These concepts were not included in the podcast or counted in the average, pass, or mastery scores.

**Table S2. Intended behaviours**

*Think about an illness that you might get. Imagine someone claiming (saying) that a particular treatment might help you get better.*

|  | **How likely are you to find out what the claim was based on (for example by asking the person making the claim)?** | | **How likely are you to find out if the claim was based on research studies comparing the treatment to no treatment (a fair comparison)?** | | **How likely are you to say “yes” if you are asked to participate in a research study comparing two treatments for your illness (a fair comparison)?** | |
| --- | --- | --- | --- | --- | --- | --- |
| **One-year follow-up** | | | | | | |
|  | **Control group**  (n=256) | **Podcast group**  (n=267) | **Control group**  (n=256) | **Podcast group**  (n=267) | **Control group**  (n=256) | **Podcast group**  (n=267) |
| Very unlikely | 11 (4.3%) | 18 (6.7%) | 16 (6.3%) | 15 (5.6%) | 10 (3.9%) | 15 (5.6%) |
| Unlikely | 27 (10.5%) | 31 (11.6%) | 37 (14.5%) | 56 (21.0%) | 18 (7.0%) | 33 (12.4%) |
| Likely | 151 (59.0%) | 145 (54.3%) | 130 (50.8%) | 106 (39.7%) | 140 (54.7%) | 104 (39.0%) |
| Very likely | 50 (19.5%) | 57 (21.3%) | 57 (22.3%) | 68 (25.5%) | 79 (30.9%) | 91 (34.1%) |
| I don’t know | 16 (6.3%) | 15 (5.6%) | 15 (5.9%) | 20 (7.5%) | 5 (2.0%) | 23 (8.6%) |
| Missing | 1 (0.4%) | 1 (0.4%) | 1 (0.4%) | 2 (0.7%) | 4 (1.6%) | 1 (0.4%) |
| **Likely or**  **very likely*** | **201 (78.5%)** | **202 (75.7%)** | **187 (73.0%)** | **174 (65.2%)** | **219 (85.5%)** | **195 (73%)** |
| **Odds ratio (95% CI)^†^** | 0.9  (0.6 to 1.3)  p=0.53 | | 0.7  (0.5 to 1.0)  p=0.07 | | 0.5  (0.3 to 0.7)  p=0.0005 | |
| **Adjusted Difference^†^** | **-2.3%**  (-10.5% to 4.4%) | | **-7.5%**  (-16.4% to 0.4%) | | **-12.6%**  (-22.3% to -4.8%) | |
| **Initial results shortly after listening to the podcast^12^** | | | | | | |

| **Likely or**  **very likely*** | **210 (76.9%)** |  | **219 (75.0%)** | **194 (71.1%)** | **217 (75.3%)** | **224 (82.1%)** | **238 (82.6%)** |
| --- | --- | --- | --- | --- | --- | --- | --- |
| **Odds ratio** |  | 0.9  (95% ci 0.6 to 1.4)  p=0.72 | | 1.2  (95% ci 0.9 to 1.8)  p=0.27 | | 1.0  (95% ci 0.7 to 1.6)  p=0.90 | |
| **Adjusted Difference** |  | **-1.3%**  (95% CI -7.9% to 6.2%) | | **4.1%**  (95% CI -3.1% to 11.6%) | | **0.4%**  (95% CI -5.7% to 6.9%) | |

* Missing values and don’t know are pooled with unlikely and very unlikely.

^†^ The odds ratios are adjusted for the stratification variables (education and child’s study group in the Informed Health Choices primary school trial). The odds ratios have been converted to differences using the control group as the reference.

**Table S3. Self-efficacy**

*How difficult or easy would you find each of these actions to be?*

|  | **Assessing whether a claim about a treatment is based on research studies comparing treatments (a fair comparison)** | | **Assessing where I can find information about treatments that is based on research studies comparing treatments (fair comparisons)** | | **Assessing how sure I can be about the results of a research study comparing treatments (the trustworthiness of the results)** | | **Assessing if the results of research studies comparing treatments are likely to be relevant to me** | |
| --- | --- | --- | --- | --- | --- | --- | --- | --- |
| **One-year follow-up** | | | | | | | | |
|  | **Control group**  (n=256) | **Podcast group**  n=267 | **Control group**  (n=256) | **Podcast group**  n=267 | **Control group**  (n=256) | **Podcast group**  n=267 | **Control group**  (n=256) | **Podcast group**  n=267 |
| Very difficult | 29 (11.3%) | 41 (15.4%) | 36 (14.1%) | 26 (9.7%) | 36 (14.1%) | 33 (12.4%) | 20 (7.8%) | 23 (8.6%) |
| Difficult | 87 (34.0%) | 79 (29.6%) | 67 (26.2%) | 75 (28.1%) | 83 (32.4%) | 93 (34.8%) | 58 (22.7%) | 62 (23.2%) |
| Easy | 105 (41.0%) | 102 (38.2%) | 104 (40.6%) | 105 (39.3%) | 88 (34.4%) | 89 (33.3%) | 107 (41.8%) | 119 (44.6%) |
| Very easy | 25 (9.8%) | 29 (10.9%) | 40 (15.6%) | 42 (15.7%) | 40 (15.6%) | 31 (11.6%) | 54 (21.1%) | 42 (15.7%) |
| I don’t know | 9 (3.5%) | 13 (4.9%) | 8 (3.1%) | 15 (5.6%) | 8 (3.1%) | 20 (7.5%) | 16 (6.3%) | 18 (6.7%) |
| Missing | 1 (0.4%) | 3 (1.1%) | 1 (0.4%) | 4 (1.5%) | 1 (0.4%) | 1 (0.4%) | 1 (0.4%) | 3 (1.1%) |
| **Easy or**  **very easy*** | **130 (50.8%)** | **131 (49.1%)** | **144 (56.2%)** | **147 (55.1%)** | **128 (50.0%)** | **120 (44.9%)** | **161 (62.9%)** | **161 (60.3%)** |
| **Odds ratio (95% CI)^†^** | 0.9  (0.6 to 1.3)  p=0.62 | | 1.0  (0.7 to 1.4)  p=0.99 | | 0.8  (0.6 to 1.2)  p=0.26 | | 0.9  (0.6 to 1.3)  p=0.64 | |
| **Adjusted difference^†^** | **-2.2%**  (-10.8% to 6.4%) | | **-0.1%**  (-8.8% to 8.3%) | | **-5.1%**  (-13.5% to 3.6%) | | **-2.0%**  (-10.7% to 6.1%) | |
| **Initial results shortly after listening to the podcast^12^** | | | | | | | | |

| **Easy or**  **very easy** | **133 (48.7%)** | **185 (64.2%)** | **155 (56.8%)** | **208 (72.2%)** | **114 (41.8%)** | **205 (71.2%)** | **182(66.7%)** | **222 (77.1%)** |
| --- | --- | --- | --- | --- | --- | --- | --- | --- |
| **Odds ratio** | 1.9  (95% CI 1.4 to 2.7)  p=0.0002 | | 2.0  (95% CI 1.4 to 2.8)  p= 0.0002 | | 3.4  (95% CI 2.4 to 4.9)  p<0.0001 | | 1.7  (95% CI 1.1 to 2.4)  p=0.008 | |
| **Adjusted difference** | **15.5%**  (95% CI 7.4% to 23.6%) | | **15.4%**  (95% CI 7.6% to 23.3%) | | **29.4%**  (95% CI 21.6% to 37.3%) | | **10.2%**  (95% CI 3.0% to 17.8%) | |

* Missing values and don’t know are pooled with difficult and very difficult.

† The odds ratios are adjusted for the stratification variables (education and child’s study group in the Informed Health Choices primary school trial). The odds ratios have been converted to differences using the control group as the reference.

**Table S4. Self-reported behaviour - awareness of treatment claims**

*How often do you hear treatment claims?*

|  | **Control group**  (n=256) | **Podcast group**  (n=267) |
| --- | --- | --- |
| One or more most days | 90 (35.2%) | 98 (36.7%) |
| One or more most weeks | 52 (20.3%) | 68 (25.5%) |
| One or more most months | 67 (26.2%) | 52 (19.5%) |
| Almost never | 28 (10.9%) | 36 (13.5%) |
| I don’t know | 17 (6.6%) | 12 (4.5%) |
| Missing | 2 (0.8%) | 1 (0.4%) |
| **One or more most days or most weeks** | **142 (55.5%)** | **166 (62.2%)** |
| **Odds ratio (one or more most days or most weeks versus other)*** | 1.4  (95% CI 1.0 to 2.0)  p=0.08 | |
| **Adjusted difference*** | **7.6%**  (95% CI -1.0% to 15.4%) | |

*The odds ratio for the dichotomised data is shown in the table. The odds ratio is adjusted for the stratification variables (education and child’s study group in the Informed Health Choices primary school trial). The odds ratio has been converted to a difference using the control group as the reference. The proportional odds ratio from the mixed ordinal logistic regression was 0.89 (95% CI 0.65 to 1.24, p=0.50).

**Table S5. Self-reported behaviour - assessment of the basis of treatment claims**

*For the last treatment claim that you heard, did you think about the basis of that treatment claim?*

|  | **Control group**  (n=256) | **Podcast group**  (n=267) |
| --- | --- | --- |
| Yes | 185 (72.3%) | 171 (64.0%) |
| No | 46 (18.0%) | 57 (21.3%) |
| Don’t remember | 22 (8.6%) | 37 (13.9%) |
| Missing | 3 (1.2%) | 2 (0.7%) |
| **Odds ratio (yes versus other)*** | 0.7  (95% CI 0.5 to 1.0)  p=0.05 | |
| **Adjusted difference*** | **-8.2%**  (95% CI -17.3% to 0.0%) | |

^*^ The odds ratio is adjusted for the stratification variables (education and child’s study group in the Informed Health Choices primary school trial). The odds ratio has been converted to a difference using the control group as the reference.

**Table S6. Self-reported behaviour - assessment of trustworthiness of treatment claims**

*How sure are you that the treatment claim you heard is true or can be trusted?*

|  | **Control group**  (n=256) | **podcast group**  (n=267) |
| --- | --- | --- |
| Not very sure because I don’t know the reason behind the claim | 54 (21.1%) | 83 (31.1%) |
| Not very sure because the reason behind the claim was not good | 23 (9.0%) | 51 (19.1%) |
| Very sure because the reason behind the claim was good | 128 (50.0%) | 66 (24.7%) |
| I don’t know because I don’t know how to decide whether it is true or not | 50 (19.5%) | 64 (24.0%) |
| Missing | 1 (0.4%) | 3 (1.1%) |
| **Very sure or I don’t know** | **178 (69.5%)** | **130 (48.7%)** |
| **Odds ratio (very sure or I don’t know vs other)*** | 0.4  (95% CI 0.3 to 0.6)  p<0.0001 | |
| **Adjusted difference*** | **-20.9%**  (95% CI -29.9% to -12.0%) | |
| **Consistent with what they identified as the basis for the claim^†^** | **50 (19.5%)** | **61 (22.8%)** |
| **Odds ratio (consistent with what they identified as the basis for the claim)** | 1.3  (95% CI 0.8 to 1.9)  p=0.30 | |
| **Adjusted difference*** | **3.8%**  (95% CI -2.9% to 12.3%) | |

^*^ The odds ratios are adjusted for the stratification variables (education and child’s study group in the Informed Health Choices primary school trial). The odds ratios have been converted to a difference using the control group as the reference.

^†^ See Table 3.

**Table S7. Self-reported behaviour - assessment of advantages and disadvantages of treatments**

*How sure are you about the advantages and disadvantages of the [most recent] treatment you used?*

|  | **Control group**  (n=256) | **Podcast group**  (n=267) |
| --- | --- | --- |
| A) Not very sure because I don’t know the reasons behind the claims about the good and bad things that treatment makes happen | 52 (20.3%) | 71 (26.6%) |
| B) Not very sure because there was not a good reason behind the claims about the advantages of the treatment | 13 (5.1%) | 29 (10.9%) |
| C) Not very sure because I only know about the advantages of the treatment. I also need to know about the disadvantages | 65 (25.4%) | 64 (24.0%) |
| D) Very sure because there is a good reason behind the claims about the advantages and disadvantages of the treatment | 101 (39.5%) | 72 (27.0%) |
| E) I did not use any treatment | 21 (8.2%) | 28 (10.5%) |
| Missing | 4 (1.6%) | 3 (1.1%) |
| **Odds ratio* for C versus any other response** | 0.9  (95% CI 0.6 to 1.4)  p=0.77 | |
| **Adjusted difference* for C versus any other response** | **-1.1%**  (95% CI -7.7% to 7.0%) | |
| **Odds ratio* for D versus any other response** | 0.5  (95% CI 0.4 to 0.8)  p=0.001 | |
| **Adjusted difference* for D versus any other response** | **-13.3%**  (95% CI -19.9% to -5.5%) | |

^*^ The odds ratios are adjusted for the stratification variables (education and child’s study group in the Informed Health Choices primary school trial). The odds ratios have been converted to a difference using the control group as the reference.

**Table S8. Subgroup analyses - education**

|  | **Control group** | **Podcast group** | | **Odds ratio*** | **Adjusted difference*** |
| --- | --- | --- | --- | --- | --- |
| **Mean score, %** | | | | |  |
| **Primary education**  (n=235) | n=112 | n=123 | |  |  |
|  | Mean score: 46.5%  (SD 19.9) | Mean score: 54.7%  (SD 20.0) | |  | **Mean difference: 8.2%**  (95% CI 3.1 to 13.3) |
| **Secondary education**  (n=152) | n=68 | n=85 | |  |  |
|  | Mean score: 56.0%  (SD 19.5) | Mean score: 58.1%  (SD 18.8) | |  | **Mean difference: 1.8%**  (95% CI -4.3 to 7.9) |
| **Tertiary education**  (n=132) | n=74 | n=58 | |  |  |
|  | Mean score: 59.2%  (SD 19.6) | Mean score: 68.9%  (SD 21.6) | |  | **Mean difference: 9.3%**  (95% CI 2.3 to 16.3) |
| Interaction: secondary versus primary | | | |  | p=0.005 |
| Interaction: tertiary versus primary | | | |  | p<0.0001 |
| **Passing score** (> 11 out of 18 correct answers) | | | | |  |
| **Primary education**  (n=235) | n=112 | n=123 |  | |  |
|  | 28.6% of parents  n=32 | 41.5% of parents  n=51 | 1.8  (95% CI 1.0 to 3.1) | | **13.1% more parents** (95% CI 0.8 to 26.9) |
| **Secondary education**  (n=152) | n=68 | n=85 |  | |  |
|  | 39.7% of parents  n=27 | 38.8% of parents  n=33 | 0.9  (95% CI 0.5 to 1.8) | | **1.8% fewer parents** (95% CI -15.7 to 14.5) |
| **Tertiary education**  (n=132) | n=74 | n=58 |  | |  |
|  | 55.4% of parents  n=41 | 70.7% of parents  n=41 | 1.9  (95% CI 0.9 to 4.0) | | **14.8% more parents** (95% CI -2.1 to 27.9) |
| Interaction: secondary versus primary | | | p=0.57 | |  |
| Interaction: tertiary versus primary | | | p<0.0001 | |  |
| **Mastery score** (> 15 out of 18 correct answers) | | | | | |
| **Primary education**  (n=235) | n=112 | n=123 | |  |  |
|  | 5.4% of parents  n=6 | 13.8% of parents  n=17 | | 2.8  (95% CI 1.1 to 8.1) | **8.5% more parents**  (95% CI 0.7 to 26.2) |
| **Secondary education**  (n=152) | n=68 | n=85 | |  |  |
|  | 14.7% of parents  n=10 | 18.8% of parents  n=16 | | 1.3  (95% CI 0.6 to 3.2) | **3.7% more parents**  (95% CI -6.0 to 20.9) |
| **Tertiary education**  (n=132) | n=74 | n=58 | |  |  |
|  | 14.9% of parents  n=11 | 32.8% of parents  n=19 | | 2.7  (95% CI 1.2 to 6.6) | **17.4% more parents**  (95% CI 2.2 to 38.5) |
| Interaction: secondary versus primary | | | | p=0.06 |  |
| Interaction: tertiary versus primary | | | | p=0.0004 |  |

*Adjusted for child’s study group. The odds ratios have been converted to differences using the control as the reference.

**Table S9. Subgroup analyses - child in school that used IHC primary school resources**

| **Child’s allocation in IHC primary school resources** | **Parent in Control group** | **Parent in Podcast group** | **Odds ratio*** | **Adjusted difference^*^** |
| --- | --- | --- | --- | --- |
| **Mean score, %** | | | | |
| **Child in control group**  (n=214) | n=109 | n=105 |  |  |
|  | Mean score: 49.3%  (SD 18.6%) | Mean score: 57.1%  (SD 20.1%) |  | **Mean difference: 8.5%**  (95% CI 3.4% to 13.6%) |
| **Child in intervention group**  (n=309) | n=147 | n=162 |  |  |
|  | Mean score: 55.1%  (SD 21.4%) | Mean score: 60.0%  (SD 20.9%) |  | **Mean difference: 5.3%**  (95% CI 0.7% to 9.9%) |
| Interaction: child in intervention vs control group | | |  | p=0.02 |
| **Passing score** (> 11 out of 18 correct answers) | | | | |
| **Child in control group**  (n=214) | n=109 | n=105 |  |  |
|  | 28.4% of parents  n=31 | 45.7% of parents  n=48 | 2.4  (95% CI 1.3 to 4.5) | **20.6% more parents**  (95% CI 6.3% to 35.6%) |
| **Child in intervention group**  (n=309) | n=147 | n=162 |  |  |
|  | 47.6% of parents  n=70 | 48.1% of parents  n=78 | 1.1  (95% CI 0.7 to 1.8) | **2.4% more parents**  (95% CI -9.0% to 13.8%) |
| Interaction: child in intervention vs control group | | | p=0.01 |  |
| **Mastery score** (>15 out of 18 correct answers) | | | | |
| **Child in control group**  (n=214) | n=109 | n=105 |  |  |
|  | 8.3% of parents  n=9 | 15.2% of parents  n=16 | 2.2  (95% CI 0.9 to 5.4) | **8.0% more parents**  (95% CI -0.7% to 24.4%) |
| **Child in intervention group**  (n=309) | n=147 | n=162 |  |  |
|  | 12.2% of parents  n=18 | 22.2% of parents  n=36 | 2.2  (95% CI 1.2 to 4.2) | **11.0% more parents**  (95% CI 1.7% to 24.5%) |
| Interaction: child in intervention vs control group | | | p=0.09 |  |

*Adjusted for parent’s education. The odds ratios have been converted to differences using the control group as the reference.

**Table S10. Effect of IHC primary school resources on parents**

|  | **IHC primary school resources** | |  |  |
| --- | --- | --- | --- | --- |
| **Parents’ scores** | **Child in control school** | **Child in intervention school** | **Odds ratio*** | **Adjusted difference*** |
| **One-year follow-up**  Mean score (%) | Mean score 53.1%  (SD 19.7%) | Mean score 57.7%  (SD 21.3%) |  | **Mean difference: 4.2%**  (95% CI 0.7% to 7.7%)  p=0.02 |
| **Initially after listening to the podcast**  Mean score (%) | Mean score 58.5%  (SD 19.9%) | Mean score 61.6%  (SD 20.3%) |  | **Mean difference: 2.6%**  (95% CI -0.5% to 5.7%)  p=0. 0.10 |
| **One-year follow-up**  Passing score | 79/214 (36.9%) | 148/309 (47.9%) | 1.6  (95% CI 1.1 to 2.4)  p=0.01 | **11.9% more parents**  (95% CI 2.8% to 21.2%) |
| **Initially after listening to the podcast**  Passing score | 121/231 (52.4%) | 185/330 (56.1%) | 1.13  (95% CI 0.79 to 1.63)  p=0.50 | **3.1% more parents**  (95% CI -5.9% to 11.8%) |
| **One-year follow-up**  Mastery score | 25/214 (11.7%) | 54/309 (17.5%) | 1.6  (95% CI 0.9 to 2.7)  p=0.09 | **5.4% more parents**  (95% CI -0.7% to 14.4%) |
| **Initially after listening to the podcast**  Mastery score | 16.5% of parents  n=38 | 21.2% of parents  n=70 | 1.37  (95% CI 0.86 to 2.17)  p=0.18 | **4.8% more parents**  (95% CI -1.9% to 13.5%) |

*Adjusted for parent’s study group (podcast) and education. The odds ratios have been converted to differences using having a child in a control school as the reference.
